# Remotely‐Sensed Game Trails Are a Behavioral Footprint That Explains Patterns of Herbivore Habitat Use

**DOI:** 10.1002/ece3.70792

**Published:** 2025-01-11

**Authors:** Keenan Stears, Melissa H. Schmitt, Mike J. Peel, Tsumbedzo Ramalevha, Douglas J. McCauley, Dave I. Thompson, Deron E. Burkepile

**Affiliations:** ^1^ Department of Biology University of North Dakota Grand Forks North Dakota USA; ^2^ Department of Ecology, Evolution, and Marine Biology University of California Santa Barbara Santa Barbara California USA; ^3^ South African Environmental Observation Network Ndlovu Node, Scientific Services, Kruger National Park Phalaborwa South Africa; ^4^ School of Biology & Environmental Sciences University of Mpumalanga Nelspruit South Africa; ^5^ Agricultural Research Council Animal Production Institute, Rangeland Ecology Group Nelspruit South Africa; ^6^ School for Animal, Plant and Environmental Sciences University of the Witwatersrand Johannesburg South Africa; ^7^ Applied Behavioural Ecology and Ecosystem Research Unit University of South Africa Florida South Africa; ^8^ Unit for Environmental Sciences and Management North‐West University Potchefstroom South Africa; ^9^ Marine Science Institute University of California Santa Barbara Santa Barbara California USA; ^10^ School of Geography, Archaeology, and Environmental Studies University of the Witwatersrand Johannesburg South Africa

**Keywords:** African savanna, game path, risk and reward trade‐off, species distributions, woody plant cover

## Abstract

Trade‐offs between food acquisition and predator avoidance shape the landscape‐scale movements of herbivores. These movements create landscape features, such as game trails, which are paths that animals use repeatedly to traverse the landscape. As such, these trails integrate behavioral trade‐offs over space and time. Here, we used remotely sensed imagery to analyze the density of game trails with spatial environmental variables to understand landscape‐scale patterns of herbivore habitat use in an African savanna. Woody plant cover was the best predictor of game trail density, with the highest densities correlating with intermediate woody plant cover. We also explored how patterns of game trail density compared to two known measures of herbivore habitat use (i.e., dung counts and maximum entropy modeling) and found strong quantitative fits. To understand the patterns revealed by the density of game trails, we explored the trade‐off between food acquisition and perceived predation risk across a woody plant cover gradient. Using behavioral observations, we found that the relationship between woody plant cover and the distribution of game trails was likely driven by the risk and reward trade‐off, with less vigilance and more feeding occurring in areas with a high density of game trails and intermediate woody cover. Ultimately, we show that game trails are a novel data source that can be used to identify broadly‐occurring patterns of herbivore habitat use over large spatial scales.

## Graphical abstract

We used remotely sensed imagery to analyze the density of game trails with spatial environmental variables to understand landscape‐scale patterns of herbivore habitat use in an African savanna. Game trails are paths that animals use repeatedly to traverse the landscape. We found that game trails are a novel data source that can be used to identify patterns of herbivore habitat use at large spatial scales.

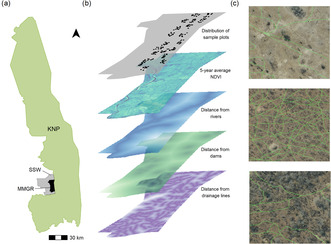

## 1 Introduction

A primary driver that influences the distribution of animals within heterogeneous landscapes is the trade‐off between the distribution of food resources and perceived predation risk (Davies et al. 2021; Gaynor et al. 2019; Stears and Shrader 2015). In many systems, herbivores frequently forage in ways that either maximize their nutrient or food intake (Fryxell, Wilmshurst, and Sinclair 2004; Wilmshurst and Fryxell 1995). To achieve this, herbivores may select for specific habitats that provide nutrient‐rich vegetation and/or high food availability. However, variation in predation risk may prevent herbivores from utilizing these resource‐rich habitats. Thus, when making decisions regarding habitat use in heterogeneous environments, herbivores often must decide whether to avoid risky habitats or tolerate increased predation risk in exchange for using resource‐rich but risky areas. The risk and reward trade‐off that prey species make between predation risk and food acquisition results in herbivores living within a landscape of fear (Davies et al. 2021; Laundré, Hernández, and Altendorf 2001). Insights from this body of work have helped inform how prey species may respond to heterogeneous landscapes and how predator–prey interactions and their impacts on ecological function may vary dynamically (Madin, Madin, and Booth 2011; Palmer et al. 2017; Stears and McCauley 2018).

Because the strengths of top‐down and bottom‐up processes are context dependent, the trade‐off between food acquisition and predation risk is modulated by habitat heterogeneity (Anderson et al. 2010; Davies et al. 2021; Valeix et al. 2010). Woody plant cover influences habitat heterogeneity while also simultaneously influencing food availability and perceived predation risk. For example, an increase in woody plant cover is frequently associated with elevated perceived predation risk for a range of ungulate species due to an increase in ambush opportunities for predators that is associated with dense woody cover (Davies et al. 2016; Underwood 1982). Consequently, browsing species (i.e., those that eat woody plants) typically avoid densely wooded habitats despite these habitats providing high food availability (Riginos 2015). Thus, habitat heterogeneity and the impacts it has on both bottom‐up and top‐down processes are key in explaining important behavioral and ecological processes that structure the spatial distribution of prey species across the landscape. Understanding the processes driving species distributions and habitat selection provides important insights into the structure and function of ecological communities and the maintenance of biodiversity (Morris 2003).

Habitat use is frequently predicted using approaches that require occurrence data, which are often obtained via GPS tracking of individuals or large‐scale camera grids (e.g., Anderson et al. 2016; Stears et al. 2019). Such approaches can be costly and time‐consuming, frequently temporally limited, and often only represent a few individuals from the population. However, the advent of open‐access satellite imagery and unmanned aerial vehicles (UAVs) can enable remote observation of how prey species use their landscape. For example, Madin, Madin, and Booth (2011) employed this approach and identified grazing halos—rings of bare substrate devoid of seaweed—surrounding coral patch reefs in Australia. The outer edge of the grazing halo, where the fish stop feeding, represents the point at which predation risk outweighs the benefits of food acquisition. Thus, these halos represent a landscape‐scale physical footprint of behavioral interactions (i.e., the trade‐off between food acquisition and perceived risk) that drive patterns of herbivore habitat use (Madin, Madin, and Booth 2011).

In terrestrial ecosystems, game trails, which are paths that animals use repeatedly to traverse the landscape, likely represent a metric of herbivore habitat use and are akin to the grazing halos of Madin, Madin, and Booth (2011) described above. Game trails can be used as foraging paths or as conduits that animals use to navigate the landscape (Agnew 1966; Shannon et al. 2009). The use of game trails as a possible measure of habitat use by large mammals was suggested by Agnew (1966) and has since been used to understand forest elephant, *Loxodonta africana cyclotis*, and savanna elephant, *Loxodonta africana*, movements (Vanleeuwé and Gautier‐Hion 1998; Shannon et al. 2009). Because game trails are formed through repeated use over time, they represent time‐averaged use of the landscape and can provide population‐level inferences (Shannon et al. 2009). Thus, they can be used to identify broadly‐occurring patterns of herbivore habitat use at large spatial scales.

Here, we aimed to use game trails detected from aerial imagery, satellite‐derived habitat data, and in situ behavioral observations to examine patterns of habitat use and risk–reward trade‐offs in an African savanna ecosystem. Our objectives were to (1) model game trail density with spatial environmental variables (i.e., woody plant cover, distance to dams/pans, distance to rivers, and distance to drainage lines) to predict community‐level herbivore habitat use at the landscape scale, (2) explore how herbivore habitat use, as predicted by game trail density, compared to two known measures of herbivore habitat use (i.e., dung counts and maximum entropy modeling), and finally (3) understand the potential drivers of the patterns revealed by the density of game trails across a heterogeneous landscape. We explored this final objective by analyzing the trade‐off between food acquisition and perceived predation risk across a woody plant cover gradient. To do this, we collected data on the antipredator behaviors of a model species—the plains zebra, *Equus quagga* —across habitats spanning a woody plant cover gradient. We hypothesized that the distribution of game trails would be able to predict herbivore habitat use and match the patterns of herbivore habitat use predicted using dung counts and maximum entropy modeling. Finally, we hypothesized that the density of game trails would reflect the risk and reward trade‐off such that high‐use areas are perceived to be safer habitats where herbivores focus relatively more on resource acquisition than predator detection and avoidance, compared to low‐use habitats.

## 2 Materials and Methods

### 2.1 Study Site

**FIGURE 1:**
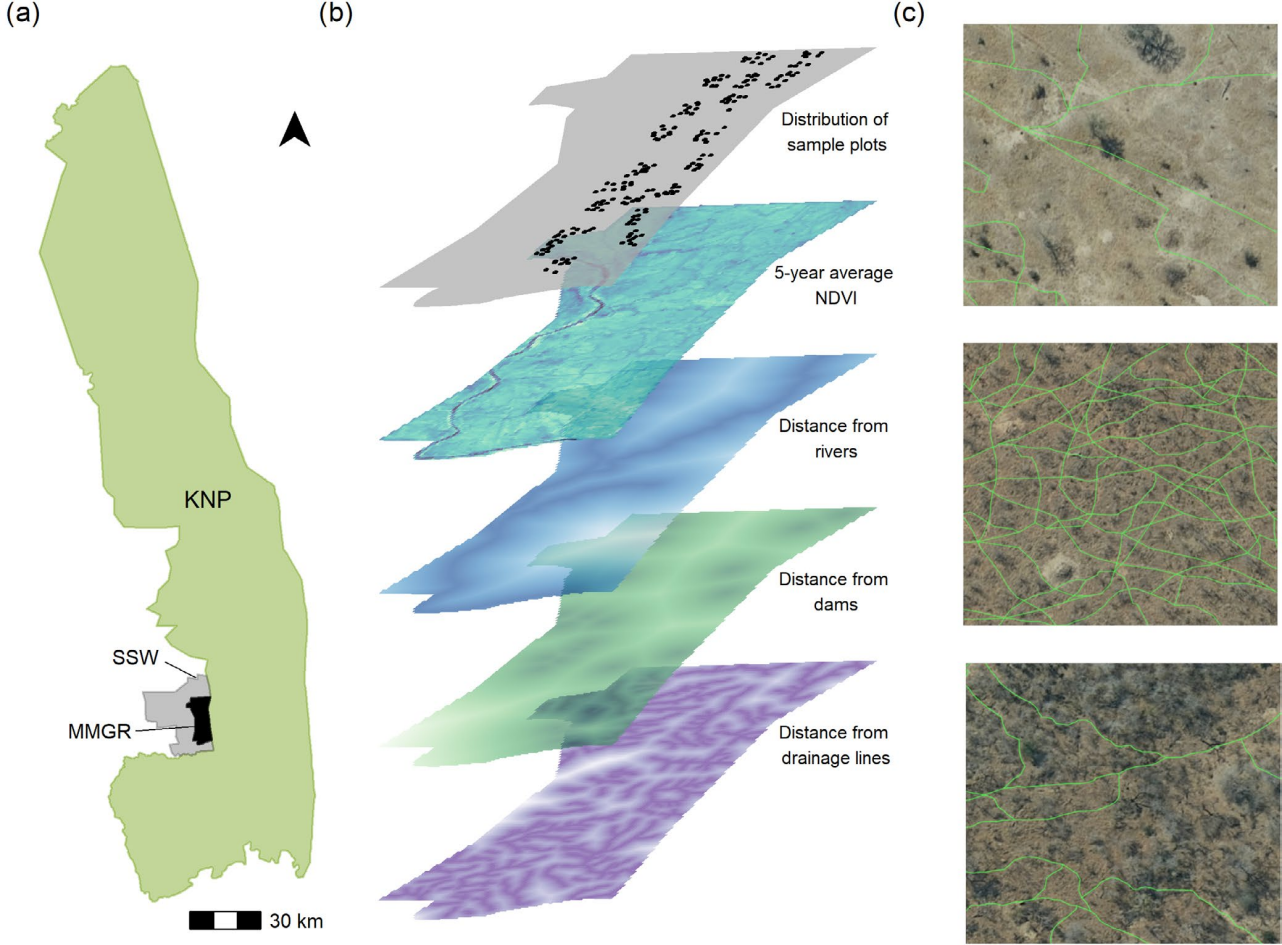
Location of our study site (Panel (a)) MalaMala Game Reserve (MMGR) in relation to the Sabi Sand Wildtuin (SSW) and the Kruger National Park (KNP). Panel (b) is a workflow schematic showing the spatial distribution of sample plots and the environmental variables that we used to model the density of game trails (m/ha) and thus, herbivore habitat use at the landscape scale. Panel (c) illustrates how the density of game trails (i.e., length of game trail: m/ha), which are illustrated by the green lines, varies across an increasing woody plant cover gradient from top to bottom in each 100 m × 100 m sample plot.

We conducted our study in the MalaMala Game Reserve (13,300 ha). MalaMala Game Reserve falls within the Sabi Sand Wildtuin–MalaMala–Sabi Sabi Private Game Reserve Complex, which forms part of the Greater Kruger National Park, South Africa (Figure 1a). Our study site is unfenced and is bordered by the Kruger National Park to the east and the Sabi Sand Wildtuin to the north, south, and west. The region's mean annual rainfall is approximately 620 mm, with summer rainfall occurring between October and March (Schulze 2008). The vast majority of mammals in the savannas of the region are present at our study site, including the entire large carnivore guild—lion ( *Panthera leo* ), leopard ( *Panthera pardus* ), cheetah ( *Acinonyx jubatus* ), African wild dog ( *Lycaon pictus* ), and hyena ( *Crocuta crocuta* )—as well as their key prey species (Radloff and du Toit 2004). For a list of herbivore species used in the analyses below, see Table S1. The vegetation at our study site is characterized by a mixed *Combretum/Terminalia* woodland (Gertenbach 1983). The Sand River is the main source of water for wildlife and flows for approximately 25 km through the study site. In addition to the Sand River, there are several small dams and pans distributed across the property. These dams and pans are fed by natural rainfall, and no artificial water has been provided on the property for the last several decades.

### 2.2 Quantifying Game Trail Density and Environmental Variables

To quantify game trail density across the landscape, we used high‐resolution aerial imagery (0.25 m resolution obtained from the CDNGI Geospatial Portal from the Department of Rural Development and Land Reform of South Africa) from the dry season of 2018 to identify individual game trails created and used by the herbivore community. From the available aerial images, we selected 200 blocks (100 m × 100 m) that were distributed across our study site (Figure 1b). These blocks spanned a range of woody plant cover as well as distances from rivers (range: ~100–4300 m). Within these 200 blocks, we hand‐traced every game trail in QGIS, and for each block, we extracted the density of game trails as measured by the total length of game trails (m) per hectare (Figure 1c). Game trails typically ranged in width from 20 to 60 cm. Given that game trails are significantly narrower than roads, we differentiated between roads and game trails based on their width. Moreover, because our study is a protected area, there are no trails left by humans or livestock.

At the landscape scale, we compiled spatial data on environmental variables that may influence herbivore habitat use. These environmental variables included woody plant density, the distance from water sources (i.e., rivers, dams, and pans), and the distance from drainage lines (i.e., a natural channel through which water/runoff concentrates and flows after heavy rains). For each of these variables, we generated a raster layer and extracted the value of each raster layer for each of the 200 blocks (see workflow outlined in Figure 1b). All spatial analyses were done using the *raster* package in R (Hijmans 2020). For the distance metrics, we calculated distance based off the centroid of each of the 200 sample blocks. All distance metrics were calculated at a 10‐m resolution (for more information on the creation of the distance layers, please see Appendix S1).

We used remotely sensed satellite data to calculate the normalized difference vegetation index (NDVI), which we used as a metric for woody plant density (strong positive correlation between NDVI and actual woody plant density: *p* < 0.001, *R* ^2^ = 0.8; see Figure S2 and text in Appendix S1). NDVI is a useful tool to link climate, vegetation, and animal distributions at large spatial and temporal scales (Pettorelli et al. 2005). Because NDVI measures the greenness of both woody and non‐woody vegetation, it can differentiate between different habitats and provides robust predictions for both tree density and cover (Kumar, Uniyal, and Lal 2007; Tsalyuk, Kelly, and Getz 2017; Wagenseil and Samimi 2007). We calculated NDVI from Sentinel‐2 satellite imagery from the peak wet season (1 January–30 April; based on long‐term rainfall data for our study site). To account for any potential effects of annual variation in rainfall on NDVI, we created a five‐year average wet season NDVI layer that represents present‐day conditions (i.e., 2015–2019) of woody plant density to combine with the game trail data obtained in 2018. We used wet season NDVI imagery to determine woody plant density because of the deciduous nature of many tree species at our study site. Ultimately, the mean NDVI layer represents the overall average woody plant density for the given period (i.e., 2015–2019) because it is unlikely that tree density will change between seasons within a year (i.e., it is not only a measure of wet season woody plant density). For more information on satellite image acquisition and processing as well as the creation of the spatial layers, please see Appendix S1.

### 2.3 Modeling Herbivore Landscape Use From Game Trails

We modeled the relationship between game trail density (i.e., herbivore habitat use) and our spatial covariates (i.e., NDVI and the distance from the nearest river, dam, and drainage line) using a generalized additive modeling (GAM) framework from the *mgcv* package in R (Wood 2017). For our GAM models, we used a cubic regression spline for each predictor variable, and we used cross‐validation to estimate the optimal amount of smoothing (Zuur et al. 2009). For each variable, we tested whether the smoother term was significant over a linear model and replaced non‐significant smoother terms with linear terms to avoid overfitting the data (Suárez‐Seoane, Osborne, and Carlos Alonso 2002). For model selection, we used the *dredge* function in the *MuMIn* package (Barton 2020) to explore all possible combinations of explanatory variables. The best‐fit model was determined using AICc and Akaike weights (Burnham and Anderson 2002). We then used the *gam.check* function to confirm model fit and diagnostics of the best fit model.

To evaluate our best‐fit model, we used cross‐validation. To do this, we randomly divided the data into a training dataset (70% of the observations) for model fitting, with the remainder of the data (30%) used as an independent test set for model validation (Joseph 2022). We determined the strength of the relationship between the observed and predicted values using a Pearson's correlation coefficient. Finally, we created our predicted herbivore habitat use map for the entire study site by projecting the predictions of our best fit model onto a raster brick of our environmental variables (i.e., NDVI, distance from rivers, distance from dams, and distance from drainage lines). For this map, each pixel (100 × 100 m) represents the predicted density of game trails (i.e., total game trail length (m) per hectare). Prior to plotting, all pixels that represented areas of surface water across the study site were excluded from the projection map to ensure accurate estimates of game trail density across the landscape.

### 2.4 Comparing Habitat Use Predicted by Game Trail Density Against Common Measures of Herbivore Habitat Use

If the density of game trails is a good predictor of herbivore habitat use, we would expect a good quantitative match between the predictions of our model and other methods of assessing habitat use. To test this, we compared predicted habitat use using game trails with habitat use measured using (1) dung counts and (2) the maximum entropy species distribution model (Maxent; Phillips, Anderson, and Schapire 2006). For more information regarding the methods for the dung counts and the maximum entropy model, as well as the comparative analyses, please see the Appendix S1.

### 2.5 Woody Plant Density and the Trade‐Off Between Perceived Risk and Food Acquisition

To understand how woody plant cover influences the trade‐off between antipredator behaviors and resource acquisition (important drivers of habitat use), we used the plains zebra as a model species. We selected zebra as our model species because (1) they occur across a wide range of habitats that span a woody plant cover gradient (Schmitt et al. 2022) and (2) they share a common predator (e.g., lion) with a wide range of other common herbivore species in our system (Hayward and Kerley 2008). To show that zebras represent the overall pattern of herbivore habitat use predicted using game trails, we modeled the relationship between zebra dung density (which was a subset of the dung count data used above) and NDVI using the same generalized additive modeling (GAM) framework described above from the *mgcv* package in R (Wood 2017). We assessed whether zebra dung counts followed a quantitatively similar relationship with overall herbivore habitat use by comparing the relationship between the model‐predicted zebra dung density and the model‐predicted game trail density across a range of NDVI values using nonlinear correlation estimates.

To determine if zebra habitat use reflects the trade‐off associated with predator avoidance and food acquisition, we determined the proportion of time zebras devoted to vigilance while foraging across a woody plant cover gradient. Locations where vigilance observations were made were randomly selected along a woody plant cover gradient. This ensured that the same location for a given woody plant cover category was not repeatedly used for behavioral observations. We defined vigilance as any head‐up behavior where zebras lifted their heads above grazing height while feeding (Kluever et al. 2008; Schmitt et al. 2014; Schmitt, Stears, and Shrader 2016; Stears et al. 2020; Underwood 1982). Because we measured the time spent vigilant during a feeding bout, the inverse of this time devoted to anti‐predator behavior reflects the time zebras devote to food acquisition. We observed individual male and female zebra during a 3‐min feeding bout to calculate individual vigilance levels (i.e., time spent vigilant). To avoid pseudoreplication and any potential sex‐specific vigilance behavior, we averaged the individual vigilance levels from all sampled individuals that occurred in the same herd to create a herd average (Liley and Creel 2008; Shrader et al. 2013). From this, we calculated the proportion of time zebras devoted to vigilance for each herd. We sampled vigilance from approximately half of the individuals in the herd to calculate the herd average (*n* = 49 herds, *n* = 155 individuals). For all observations of individuals within a herd, we ensured that there were no changes in the social (e.g., herd size) or environmental (e.g., habitat) factors that could influence vigilance for that herd. Moreover, we only sampled from herds where all individuals were within a given habitat type and never from herds where individuals spanned multiple habitats (e.g., across ecotones). We observed all zebras, using binoculars, within 50 m of a stationary vehicle with the ignition turned off to limit possible effects of our presence on zebras behavior (Jørgensen, Stears, and Schmitt 2024). If any vigilance behavior was directed towards our vehicle, we discarded that vigilance observation.

During vigilance observations, it was not always possible to obtain the GPS location to extract NDVI values (i.e., woody plant density). Thus, we grouped vigilance observations into broad habitat categories characterized by differing woody plant cover (i.e., grassy savanna, semi‐open savanna, woody savanna, closed‐canopy savanna, and thicket savanna) and extracted the NDVI ranges for each habitat post hoc. We analyzed the proportion of time zebras devoted to vigilance across habitats with different woody plant densities using a generalized linear model (binomial distribution and logit‐link function) and used Tukey's post hoc analyses to determine in which habitats zebras had the lowest levels of vigilance. To control for herd size, we included the number of zebras in each herd as a covariate. We checked model assumptions using the *performance* package (Lüdecke et al. 2021). In addition, we determined how woody plant cover influenced the proportion of aborted vs. successful feeding bouts. We defined an aborted feeding bout when a zebra stopped feeding almost immediately during the 3‐min observation and moved out of a given habitat (i.e., the zebra perceived this habitat to be too risky to feed). Because mixed‐species herding can strongly influence perceived predation risk of herbivores (Schmitt et al. 2014; Schmitt, Stears, and Shrader 2016; Stears et al. 2020), we only collected data from single‐species herds that did not contain juveniles.

## 3 Results

### 3.1 Using Game Trails to Assess Herbivore Habitat Use at the Landscape Scale

**FIGURE 2:**
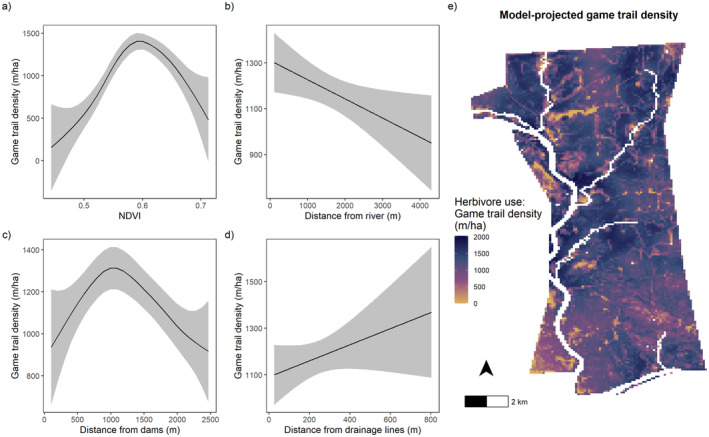
Model‐predicted patterns (mean ± 95% CI) between spatial covariates and the density of game trails (panels a–d) measured as the length of game trails per hectare (i.e., herbivore use). Panel (e) represents the model‐predicted patterns projected onto a raster brick of spatial covariates to represent herbivore habitat use across our study site as measured by game trails (m/ha). Dark colors represent areas predicted to be high use, and light colors represent areas predicted to be low use. The white pixels within our study site represent surface water (i.e., rivers and dams/pans).

Evaluation of our model revealed that our best‐fit model fit the data well and was able to predict herbivore habitat use as measured by the density of game trails (Pearson's correlation coefficient between observed and predicted values, *r* = 0.72). The best‐fit model contained all spatial covariates, with the distance from rivers and the distance from drainage lines being the two non‐smoothed variables (Table S2). The density of game trails was significantly influenced by NDVI (*χ* ^2^ = 89.03, edf = 4.230, *p* < 0.001; Figure 2a), the distance away from rivers (*z* = −2.662, df = 1, *p* = 0.008; Figure 2b), the distance from dams (*χ* ^2^ = 18.27, edf = 3.132, *p* = 0.001; Figure 2c), and the distance away from drainage lines (*z* = 2.015, df = 1, *p* = 0.04; Figure 2d). NDVI was the strongest predictor of game trail density, which had a clear unimodal response to increasing woody plant density (Table S2). The peak density of game trails was observed between NDVI values of ~0.57–0.62, which corresponds with a woody savanna (i.e., intermediate woody density) in our study system. Furthermore, the lowest densities of game trails were at either end of the NDVI spectrum, which corresponds with open grassy savannas and densely wooded thicket savannas (Figure 2a). Using the predictions from our best‐fit model, we mapped predicted herbivore habitat use (as measured by game trail density) across our study site to illustrate the spatial distribution of these patterns (Figure 2e).

### 3.2 Comparing Habitat Use Predicted by Game Trail Density Against Common Measures of Herbivore Habitat Use

#### 3.2.1 Game Trails Versus Dung Counts

**FIGURE 3:**
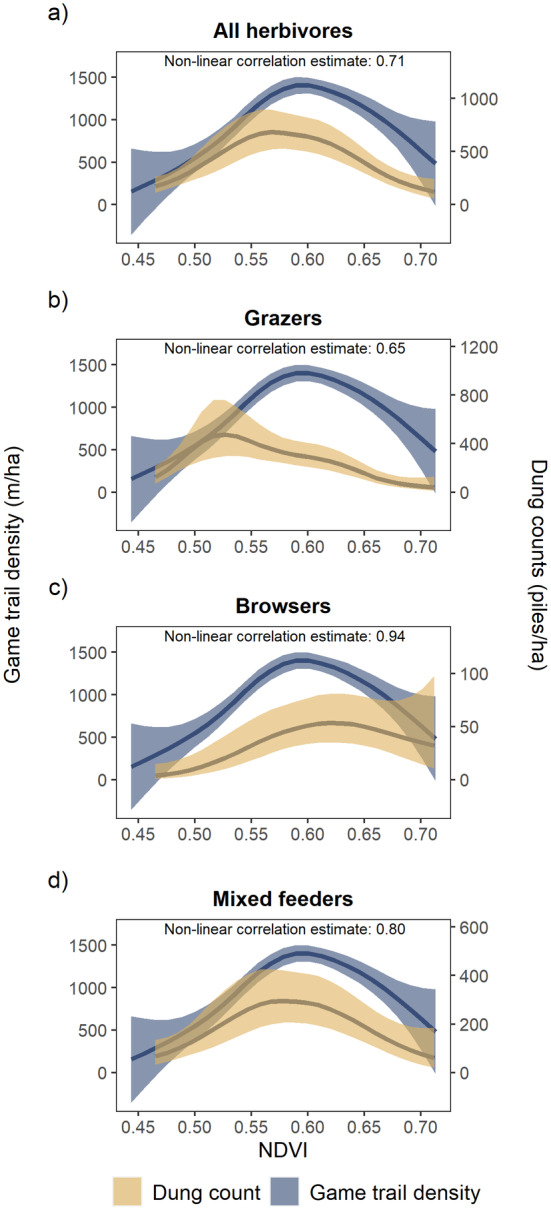
The relationship (mean ± 95% CI) between dung counts and NDVI for (a) all herbivores, (b) grazers, (c) browsers, and (d) mixed feeders overlaid with the relationship between game trail density and NDVI. The non‐linear correlation estimates show that dung density (for all herbivores combined and at the guild level) is highly correlated with the density of game trails across a woody plant cover gradient. Note the difference in scale for the dung count on the secondary axis.

We found that the model‐predicted density of game trails was highly correlated with herbivore dung density for all herbivores combined and each of the different herbivore feeding guilds (non‐linear correlation estimate for all herbivores: 0.71, *p* < 0.001; browsers: 0.94, *p* < 0.001; grazers: 0.65, *p* < 0.001; mixed feeders: 0.80, *p* < 0.001; Figure 3a–d). Dung densities for all herbivores had a significant unimodal response with NDVI (*F* = 10.75, edf = 3.437, *p* < 0.001) and peak dung densities were found in an overlapping NDVI range that had the highest game trail density (i.e., woody savannas with NDVI values ranging from 0.55 to 0.61; Figure 3a). Much like game trails, habitats on either end of the woody plant cover gradient (i.e., open savannas; NDVI: 0.44–0.49 and thicket savannas; NDVI: 0.7–0.73) had the lowest dung densities. Similarly, we found that dung densities for the different feeding guilds were also influenced by NDVI and followed similar unimodal responses (Grazers: *F* = 6.197, edf = 4.059, *p* < 0.001; Browsers: *F* = 4.095, edf = 2.606, *p* = 0.009; Mixed feeders: *F* = 5.071, edf = 2.861, *p* = 0.003).

#### 3.2.2 Game Trails Versus a Maximum Entropy Species Distribution Model

When we compared the spatial distribution of herbivore habitat use, our model using game trail density and the Maxent model both predicted similar levels of herbivore habitat use across 60% of the landscape (Figure S5). When compared to the Maxent model, our model using game trail density over‐ and underestimated herbivore habitat use across 8% and 32% of the landscape, respectively. The model using game trail density mainly underestimated herbivore use around river systems and overestimated use in areas away from rivers (Figure S5b).

### 3.3 Woody Plant Density and the Trade‐Off Between Perceived Risk and Food Acquisition

**FIGURE 4:**
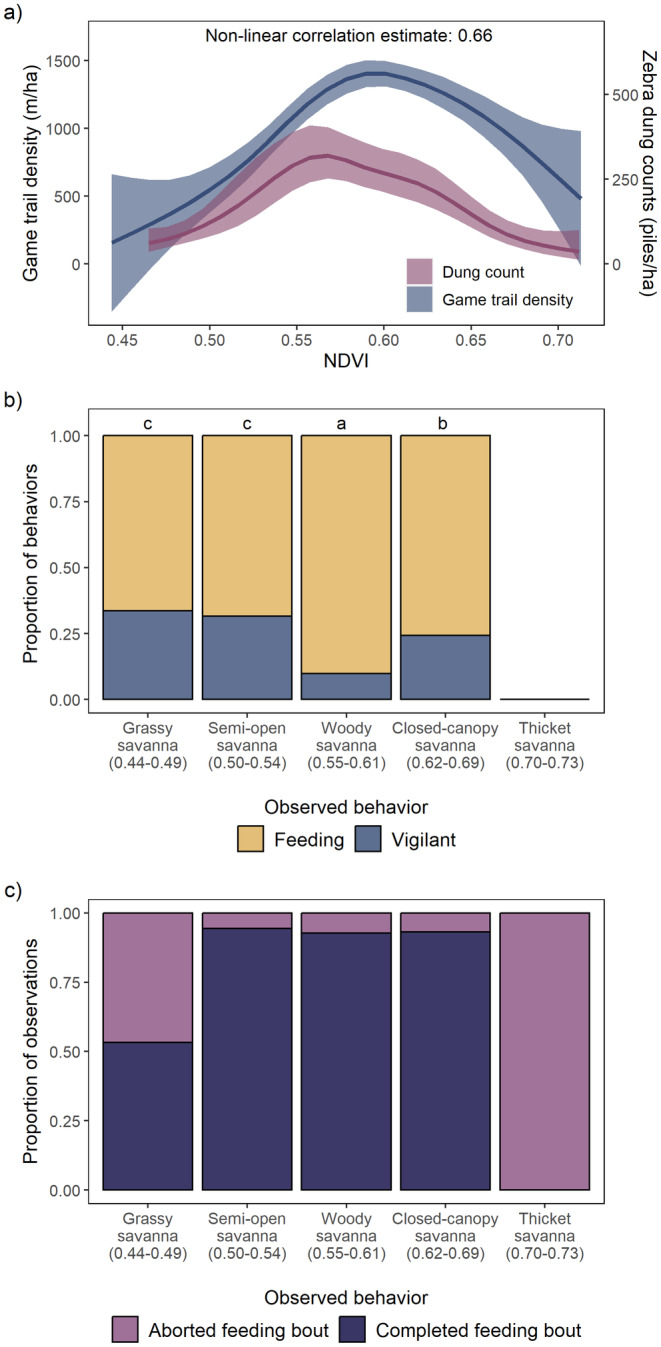
The relationship (mean ± 95% CI) between zebra dung counts and NDVI overlaid with the relationship between game trail density and NDVI (Panel (a)). The non‐linear correlation estimates show that zebra dung density is highly correlated with the density of game trails across a woody plant cover gradient. Panel (b) shows how the proportion of time devoted to food acquisition versus vigilance behavior changes across habitats along a woody plant cover gradient. Means with no letter in common are significantly different (*α* = 0.05). Panel (c) depicts the proportion of aborted feeding bouts (i.e., vigilance observations) in each habitat. Note that all observations in thicket savannas resulted in aborted feeding bouts, which explains the lack of vigilance data in panel (b) for thicket savannas. In panels (b) and (c), the NDVI range for each habitat is listed below the habitat name.

For zebras, woody plant density significantly influenced habitat use as measured by dung counts (*F* = 6.58, edf = 6.036, *p* < 0.001; Figure 4a). For a given NDVI value, zebra dung counts were significantly correlated with the density of game trails (non‐linear correlation estimate: 0.66) and therefore followed a quantitatively similar relationship with NDVI when compared to the density of game trails. Peak dung counts for zebra occurred between NDVI values that range between ~0.54 and 0.58, which mostly represent woody savanna habitats. Woody plant cover also influenced the proportion of time zebras devoted to vigilance compared to feeding (*χ* ^2^ = 815.33, df = 3, *p* < 0.001; Figure 4b). In high‐use habitats (e.g., woody savannas), zebras spent less time being vigilant and focused more on feeding compared to habitats on either end of the woody plant cover gradient. By contrast, in low‐use areas (e.g., grassy savannas), zebras invested more time being vigilant compared to feeding. We were not able to measure vigilance in low‐use thicket savannas because zebras aborted all feeding bouts within 3 min and moved out of this habitat quickly, indicating high perceived predation risk (Figure 4c).

## 4 Discussion

Visual landscape features such as the grazing halos of Madin, Madin, and Booth (2011) can be used to understand ecological processes. Here, we show that a visual landscape feature—game trails—are a landscape‐scale footprint that represents the behavioral decisions of herbivores and can be used as a novel approach to model herbivore habitat use. We show that this approach can be applied to identify broadly‐occurring patterns of herbivore habitat use for the herbivore community as a whole, as well as at the guild level.

Herbivore habitat use predicted by the density of game trails matched the predictions of two other different types of models well. For example, herbivore habitat use predicted by the density of game trails agreed with the Maxent model on predicting herbivore distribution across 60% of the landscape. The areas of disagreement between the game trail‐derived predictions of habitat use and the Maxent model are likely an artifact of the way the different data were collected (i.e., instantaneous aerial surveys for the Maxent model vs. time‐averaged for the game trails) and not due to limitations of our modeling approach. The fact that the patterns predicted by the density of game trails were a strong quantitative match to those found using dung counts (non‐linear correlation estimate: 0.71), which is another measure of herbivore habitat use that uses time‐averaged data (i.e., comparing time‐averaged versus time‐averaged metrics), suggests the use of game trails to model herbivore habitat use is robust. Thus, the differences in the way the data were collected (i.e., time‐averaged versus instantaneous) between the model using game trail density and the Maxent model likely influenced the environmental variables driving observed patterns. For example, the importance of distance from rivers observed in the Maxent model (Figure S4) is likely due to the census data being collected during the peak of the dry season at a time of day when animals are drinking from the river. This is not surprising because, frequently, models that use instantaneous count data result in different outputs when compared to models that use other techniques to monitor wildlife populations (Ndaimani et al. 2017; Redfern et al. 2002).

Environmental variables play an important role in influencing the dynamic balance between finding food and avoiding predators, which ultimately shapes how prey use their landscape (Owen‐Smith 2015). Our study reinforces this concept using a novel technique to show that the observed patterns of herbivore habitat use are similar to other studies that use more traditional methods. For example, the density of game trails (i.e., herbivore habitat use) in relation to water and drainage lines matched the patterns of herbivore habitat use found in other studies (Hopcraft, Sinclair, and Packer 2005; Ogutu et al. 2014). Moreover, we found that woody plant cover was the best predictor of game trail density because it influences both food availability and perceived risk, which is similar to the findings of other studies that identified woody plant cover as a driver of herbivore habitat use (e.g., Altendorf et al. 2001; Burkepile et al. 2013; Davies et al. 2021). Specifically, we found a unimodal relationship between game trail density and woody plant cover. Similar to our findings, a unimodal pattern of use across a woody cover gradient was observed for the herbivore community in this region by Schmitt et al. (2022), whereby habitats at either end of the woody plant cover gradient (i.e., open, grassy savannas and closed‐canopy savannas) contained low richness and abundance of herbivores. The low abundance of herbivores in these habitats is likely contributing to the low density of game trails observed in these habitats.

Further, we found that the unimodal relationship between game trail density and woody plant cover matches a key mechanistic driver (i.e., risk and reward trade‐off) of herbivore habitat use. Woody plant cover influences habitat heterogeneity while simultaneously influencing food availability and perceived predation risk. Within this trade‐off, the relative importance of finding food versus avoiding predators is highly dynamic. In our study, we found that open and semi‐open savanna habitats were predicted to be low use as measured by low game trail density. Additionally, the patterns revealed by our dung counts, which measure use across various behaviors such as feeding and resting, also show that open and semi‐open savanna habitats are low use. It is plausible that these areas are low use because they may provide low‐quality vegetation and/or have low food availability. Nevertheless, even if these habitats provide poor food resources, they could still be important habitats that are safe and provide refuge for resting. However, both game trail density and dung counts suggest that this is not the case. Instead, it is plausible that open habitats may be low‐use due to having high perceived risk, and our results support the notion that the costs of perceived risk may outweigh the benefits associated with these habitats. We found that our model species, zebra, devoted significantly more time towards antipredator behaviors (i.e., vigilance) in low‐use open and semi‐open savanna habitats. Open habitats (i.e., grassy savannas) are likely perceived to be risky by prey because cursorial predators (e.g., cheetah, spotted hyena, and African wild dog) prefer these habitats given that they do not rely heavily on woody structure to hunt (Schmidt and Kuijper 2015; Watts and Holekamp 2007). Moreover, in large‐scale open areas, there is a greater probability of being detected by a predator compared to habitats with more woody plant structure (Wheatley et al. 2021). Similarly, dense habitats, such as closed‐canopy and thicket savannas, were also predicted to be low‐use by both game trail density and dung counts, despite these habitats having high food availability for browsers and mixed‐feeders. Within these dense habitats, our model species devoted significantly more time towards antipredator behaviors (i.e., vigilance), which is consistent with other studies that show that densely‐wooded habitats are perceived to be risky because of increased ambush opportunities for predators as well as decreased escape probabilities for the prey (Davies et al. 2016; Hopcraft, Sinclair, and Packer 2005). Our results suggest that the low use of densely wooded habitats is primarily driven by perceived risk overriding food acquisition. Finally, both game trail density and dung counts identified high‐use habitats as those with intermediate woody plant cover (e.g., woody savannas), in which our model species devoted the least amount of time towards vigilance compared to habitats on either end of the woody plant cover gradient. It is likely that habitats with intermediate woody plant cover provide the best balance between food acquisition and perceived predation risk, which ultimately may explain their high use.

The mammal community in African savannas is typically represented by several herbivore feeding guilds (i.e., grazer, browser, and mixed feeder) and a diverse predator assemblage (Radloff and du Toit 2004), which further adds to the dynamic nature of these systems. Because of this diversity, generalizable patterns of how different species respond to risk may be difficult to detect and may not be easily predictable (Cusack et al. 2020; Davies et al. 2021). When using game trails as a predictor of habitat use, we are unable to determine the identity of the species using specific game trails. Thus, we linked dung counts at the guild level with game trail density along a woody plant cover gradient. We found a strong quantitative fit across all feeding guilds (non‐linear correlation estimate: browsers = 0.94; mixed feeders = 0.80; grazers = 0.65). While we find a slight mismatch in the location of peak herbivore use across a woody plant cover gradient amongst the three guilds, all guilds responded similarly to both ends of the woody plant cover gradient. Given our findings, we posit that perceived risk is the most likely factor driving the low use of these habitats, which suggests that all guilds respond similarly to the risk and reward trade‐off and how it shapes their habitat use. This pattern is not surprising given the combined high spatial and dietary overlap of both key predators (lions and leopards) within the greater study area (Balme et al. 2017; Hayward and Kerley 2008) as well as their similar use of woody cover to ambush their prey (Hayward et al. 2006; Hayward and Kerley 2005).

A potential concern with game trail data is that it is plausible that their formation and/or detection is influenced by habitat type. For example, the low density of game trails in open areas could possibly be a consequence of these habitats allowing for more diffuse travel (i.e., game trails are not easily formed) or that the game trails follow the path of least resistance to important resources. Similarly, the low density of game trails in densely wooded habitats could be due to the inability to detect game trails in densely wooded habitats. However, when we compared dung counts and vigilance observations in areas with different densities of game trails, we confirmed that large open areas and densely wooded habitats that have a low density of game trails are in fact low use by herbivores. Thus, the observed patterns of game trail density are unlikely to be driven by differences in the formation or detectability of game trails across habitats. Moreover, when using the game trail approach, it is important to understand the dynamics of game trail persistence within a focal system. Understanding these dynamics will ensure that the distribution of game trails depicts current rather than past patterns of herbivore habitat use. In our system, we observed that the majority of game trails disappeared within 2 years if they were no longer used. Finally, using game trails to quantify herbivore habitat use does not incorporate how herbivores may use other linear features (e.g., roads) as movement conduits.

Our work highlights a powerful approach to understanding animal ecology in terrestrial ecosystems. The value of using remotely sensed game trails as a data source is that one can identify broadly‐occurring patterns, ranging from the overall herbivore community level to the feeding guild level, to uncover general patterns of herbivore habitat use. Using data on the distribution of game trails as an approach to gain community‐level insights regarding patterns of habitat use is a transferable approach to other systems and contexts. Moreover, the applicability of this approach can be further extended because the distribution of game trails across the landscape can be obtained from high‐resolution satellite imagery, fixed‐wing aerial imagery, or UAV imagery. Thus, beyond its application in African savannas, game trails can be detected in other biomes (e.g., grasslands) where there is not continuous canopy cover. However, a dense canopy does not preclude the use of game trails in quantifying herbivore habitat use, but rather the method in which game trails can be measured. For example, the distribution of game trails can be mapped on foot using a hand‐held GPS (e.g., Shannon et al. 2009). Additionally, due to the long timeseries of available images for fixed‐wing and satellite data, linking game trail density to environmental variables can be used to reconstruct past wildlife‐habitat dynamics, which can act as a baseline to determine how changes in resource availability or predation risk may influence patterns of herbivore habitat use (e.g., Shannon et al. 2009). Understanding these relationships is critically important because climate‐ and human‐induced changes to landscapes and their wildlife may influence the spatial distributions of herbivores by modulating the landscape of fear (e.g., Riginos 2015; Welch et al. 2022). Finally, we show that game trails gleaned from remotely sensed images are a novel approach to measure herbivore habitat use and are able to do so because they represent the behavioral decisions of herbivores across the landscape.

## Author Contributions

**Keenan Stears:** conceptualization (equal), formal analysis (lead), funding acquisition (lead), investigation (equal), methodology (equal), project administration (equal), visualization (equal), writing – original draft (lead). **Melissa H. Schmitt:** conceptualization (equal), data curation (lead), formal analysis (supporting), funding acquisition (lead), investigation (equal), methodology (equal), project administration (equal), visualization (equal), writing – original draft (supporting). **Mike J. Peel:** data curation (lead), methodology (equal), writing – review and editing (equal). **Tsumbedzo Ramalevha:** data curation (equal), writing – review and editing (equal). **Douglas J. McCauley:** supervision (equal), writing – review and editing (equal). **Dave I. Thompson:** project administration (equal), supervision (equal), writing – review and editing (equal). **Deron E. Burkepile:** conceptualization (supporting), project administration (equal), supervision (equal), writing – review and editing (equal).

## Conflicts of Interest

The authors declare no conflicts of interest.

## Declaration

We confirm that all authors have seen and approved this version of the manuscript. All authors have substantially contributed to the work, and all persons entitled to co‐authorship have been included.

## Supporting information


Appendix S1.

## Data Availability

All data can be found at the following Zenodo Data Repository (Stears et al. 2024).
