# Supplementary material for: Remotely‐Sensed Game Trails Are a Behavioral Footprint That Explains Patterns of Herbivore Habitat Use
Source: Ecol Evol. 2025 Jan 11;15(1):e70792. doi: 10.1002/ece3.70792 (PMC11724211; doi:10.1002/ece3.70792)
Supplement: Supplementary file 1 — Appendix S1. [file ECE3-15-e70792-s001.docx]

**Supplementary material:**

**Remotely-sensed game trails are a behavioral footprint that explains patterns of herbivore habitat use**

**Table S1:** List of mammalian herbivores species and their feeding guild association following (Estes, 1992; Skinner & Chimimba, 2005). This list represents the herbivore species used in the dung counts and census data analyses.

| **Species** | **Common name** | **Feeding guild** |
| --- | --- | --- |
| *Giraffa camelopardalis* | Giraffe | Browser |
| *Sylvicapra grimmia* | Common duiker | Browser |
| *Tragelaphus angasii* | Nyala | Browser |
| *Tragelaphus scriptus* | Bushbuck | Browser |
| *Tragelaphus strepsiceros* | Kudu | Browser |
| *Ceratotherium simum* | White rhinoceros | Grazer |
| *Connochaetes taurinus* | Blue wildebeest | Grazer |
| *Equus quagga* | Plains zebra | Grazer |
| *Phacochoerus africanus* | Warthog | Grazer |
| *Syncerus caffer* | African buffalo | Grazer |
| *Aepyceros melampus* | Impala | Mixed-feeder |
| *Loxodonta africana* | African elephant | Mixed-feeder |
| *Raphicerus campestris* | Steenbok | Mixed-feeder |

The relationship between NDVI and woody plant density

To demonstrate that variation in NDVI in our study system is driven by differences in woody plant density, we correlated on-the-ground woody plant density (number of trees per hectare; N = 48 plots selected randomly; 25 x 25 m; Fig. S1a) with the extracted NDVI value for each of these sampled plots. We found a strong positive correlation between NDVI and actual woody plant density (*P* < 0.001, *R*^2^=0.8; Fig. S2), which confirms that NDVI is a good predictor of woody plant density at our study site. Based on habitat classifications using tree densities provided in Schmitt et al. (2022), we found that NDVI values of 0.44–0.49 reflected open savannas, 0.50–0.54 reflected semi-open savannas, 0.55–0.61 reflected woody savannas, 0.62–0.69 reflected closed-canopy savannas, and 0.70–0.73 reflected thicket savannas.


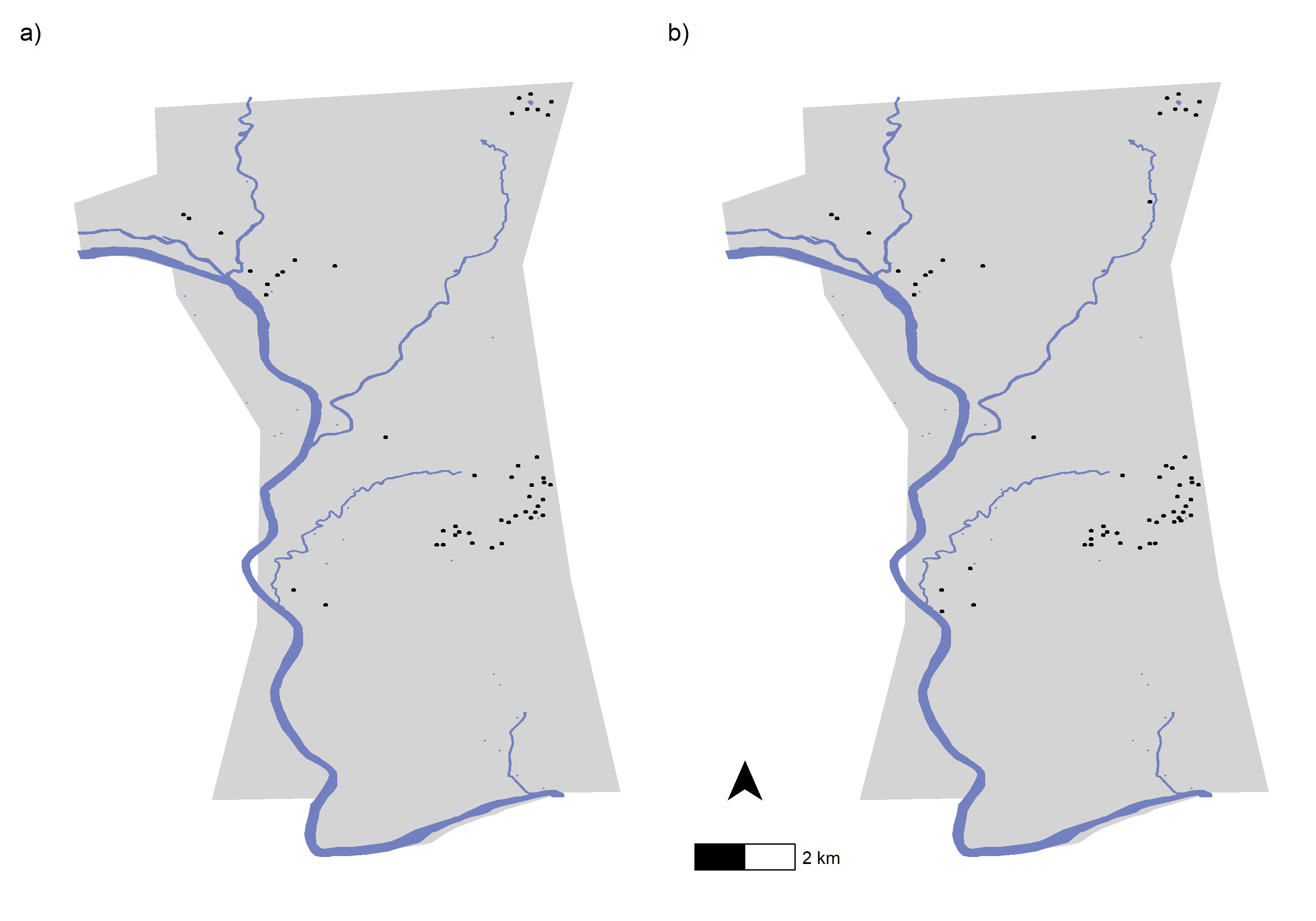


**Fig. S1:** Spatial distribution of (a) the 48-sample plots (25 x 25 m) across our study site where we enumerated all woody plants and extracted the 5-year average NDVI of each plot and (b) the 56-sample plots (25 x 25 m) across our study site where we identified and enumerated all herbivore dung piles and extracted the 5-year average NDVI of each plot.


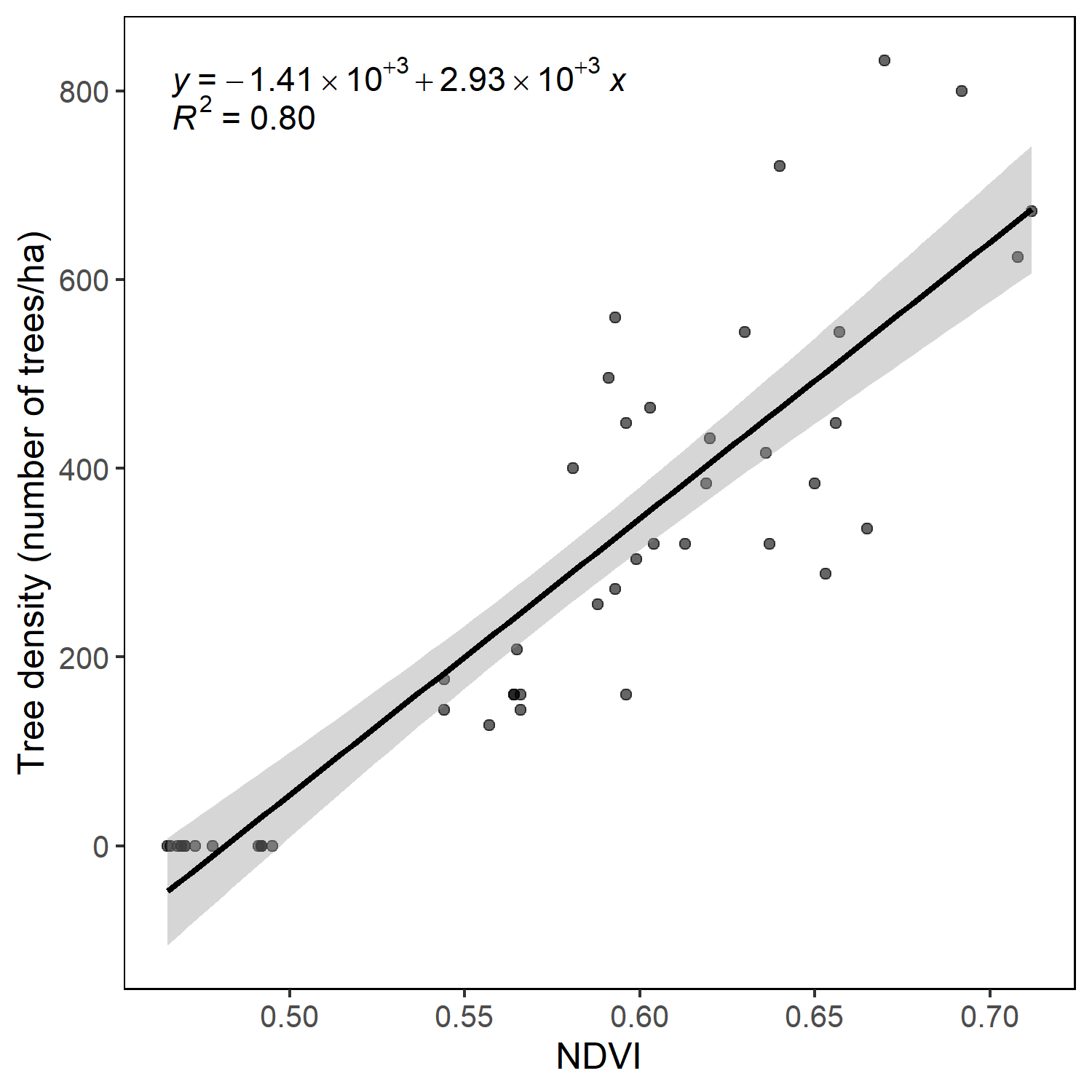


**Fig.S2:** Relationship between NDVI and tree density showing that NDVI is a good predictor of tree density.

Satellite image acquisition, processing, and the creation of the spatial layers used to model the density of game trails at the landscape scale

To calculate NDVI, we used Sentinel-2 satellite imagery. We downloaded Sentinel-2 MSI Level-1C products from the Copernicus Open Access Hub (https://scihub.copernicus.eu/) and applied the DOS1 atmospheric correction algorithm to the images. We selected cloudless images from the peak wet season (1 January–30 April), which was based on long-term rainfall data for our study site (rainfall data obtained from the South African Weather Services). To account for the effects of annual variation in rainfall on NDVI, we created a five-year average NDVI raster layer that represents current day conditions (i.e., 2015–2019) of woody plant density (NDVI is a good predictor of woody plant density, see Fig. S2). From this raster layer, we extracted the average NDVI value for each of the 200 blocks.

The location of rivers and dams were determined from remotely-sensed satellite imagery, and the location of drainage lines and pans were recorded on a hand-held GPS unit while walking the extent of these features. Ultimately, each of the 200 sample blocks has a value for game trail density (length of game trails, m/ha), an average NDVI value (-1 to 1), as well as distance values for the nearest river (m), dam (m), and drainage line (m). Finally, to map predicted game trail density (i.e., herbivore habitat use) at the landscape level, we created a raster brick of our spatial covariates and used the aggregate function to decrease the spatial resolution from 10- to 100-m.

Comparing habitat use predicted by game trail density against common measures of herbivore habitat use

*Game trails versus dung counts*

Quantifying dung densities is a common approach that is useful to predict herbivore habitat use (Barnes, 2001; Burkepile et al., 2013). Like game trails, dung counts represent a time-averaged approach to measuring herbivore use. Thus, if game trails are a good predictor of herbivore habitat use, we would expect a good quantitative match between these two methods of measuring herbivore habitat use such that areas with a high density of game trails should also have high dung counts. To test this, we first calculated dry season dung density (the number of dung piles/ha) across a gradient of woody plant density (N = 56 plots randomly selected; 25 x 25 m; see Fig. S1b). Within each plot, we identified and enumerated all herbivore dung piles, which were grouped by feeding guild (i.e., grazer, browser, and mixed-feeder), and extracted the 5-year average NDVI of each plot from the raster layer created above. Second, we assessed the relationship between overall herbivore dung density and a woody plant density gradient using a generalized additive model (GAM). Additionally, we ran separate GAM models to assess the relationship between guild-level (i.e., grazer, browser, and mixed-feeder) herbivore dung density and a woody plant density gradient. The above models included a tweedie distribution to account for a mixture of zero and positive values and, as above, we used a cubic regression spline on the predictor variable and used cross-validation to estimate the optimal amount of smoothing (Zuur et al., 2009). Finally, we tested whether game trails are a good predictor of herbivore habitat use by comparing the relationship between the model-predicted game trail density and the model-predicted dung density across a range of NDVI values using nonlinear correlation estimates. We repeated this process to assess the relationship between model-predicted game trail density and herbivore dung density for each of the herbivore feeding guilds. All dung counts were counted by the same two observers.

*Game trails versus a Maximum Entropy species distribution model*

We compared herbivore habitat use predicted by our model using game trails with another, well known, method for predicting species occurrences – the Maximum Entropy algorithm (Maxent; Phillips et al., 2006). We used Maxent because it uses presence-only occurrence data and has been shown to outperform many other species distribution models (Elith & Graham, 2009; Merow et al., 2013). Spatially-explicit herbivore occurrence data were obtained from annual aerial surveys conducted during the dry season at our study site. For these surveys, a helicopter flies the same transects every year across the entire study site and all encountered animals are enumerated (see Leeuwis et al., 2018 for details about the aerial surveys).

We used herbivore census data from 2015–2019 to correspond with the temporal scale of environmental variables used in the model using game trails. To make the outputs (i.e., spatial predictions of herbivore occurrence) of the model using game trails and the Maxent model comparable, we used the same environmental data raster brick (i.e., 5-year average NDVI as a metric of tree cover, distance from rivers, distance from dams, and the distance from drainage lines). Thus, we did not consider which environmental variables were optimal for the Maxent model, but rather included all environmental variables in the model. For the herbivore census data, we avoided spatial autocorrelation by thinning the data to only use a single occurrence per grid (100m x 100m). Furthermore, we removed all herbivore locations that fell within river beds and locations that fell within a 50 m buffer around all surface water (i.e., rivers and dams/pans). We removed these locations because they represent herbivores that are actively drinking. For the Maxent model, we used a convergence threshold of 10^-6^, 5000 iterations, and a logistic model so that predicted herbivore occurrence estimates ranged from zero (low probability of occurrence) to one (high probability of occurrence). We ran our model using cross-validation where we randomly selected 70% the herbivore census data as a training dataset and used the remaining 30% of the data for testing the resulting model (Abade et al., 2014; as per: Matawa et al., 2012; Su et al., 2021; Zhang et al., 2019). For model evaluation, we randomly generated 5000 background samples, or pseudoabsences, within the extent of our study site. We used the area under the curve (AUC) of the receiver operating characteristics (ROC) plot to assess model fit (Phillips & Dudík, 2008). In general, if the AUC value is less than or equal to 0.5, then the predictions of the model are random. However, if the value is greater than 0.5, then the predictions are better than random. We conducted all analyses using the *dismo* package in R with Maxent version 3.4.3 (Hijmans et al., 2021). Ultimately, the Maxent model was able to distinguish between suitable and unsuitable habitat for herbivores because the area under the curve (AUC) for the training data was 0.714 (Fig. S3), which is greater than a random prediction line (0.5). The distance away from rivers was the best predictor of species distributions for the Maxent model (Fig. S4).

We compared the spatial agreement in predicted herbivore habitat using game trails and the Maxent model to determine whether game trails are a good predictor of herbivore habitat use at the landscape scale (Fig. S5). To do this, we first used the maximum test sensitivity plus specificity threshold generated from the Maxent model to transform the continuous result (i.e., probability of occurrence) into a binary product (Liu et al., 2016). We then used the mean density of game trails (1124 m/ha) as a threshold to categorize herbivore habitat use into a binary measure of high- and low-use areas based on predicted game trail density (e.g., areas classified as low use had game trail densities that were lower than the mean game trail density of 1124 m/ha). We then extracted the value for each pixel (100 x 100 m) for both methods and determined model agreement based on the following metrics. We considered game trails to be a good predictor of herbivore habitat use if both approaches predicted the same value for a given pixel (i.e., either low: low or high: high). We then deemed game trails to underestimate herbivore use if the predicted value was lower than the value predicted by the Maxent model and deemed it to overestimate herbivore use if the predicted value was higher than the value predicted by the Maxent model. We then determined the proportion of pixels that fell into each of these categories. For both models, pixels that fell within river courses and pixels with surface water were excluded for this comparison.


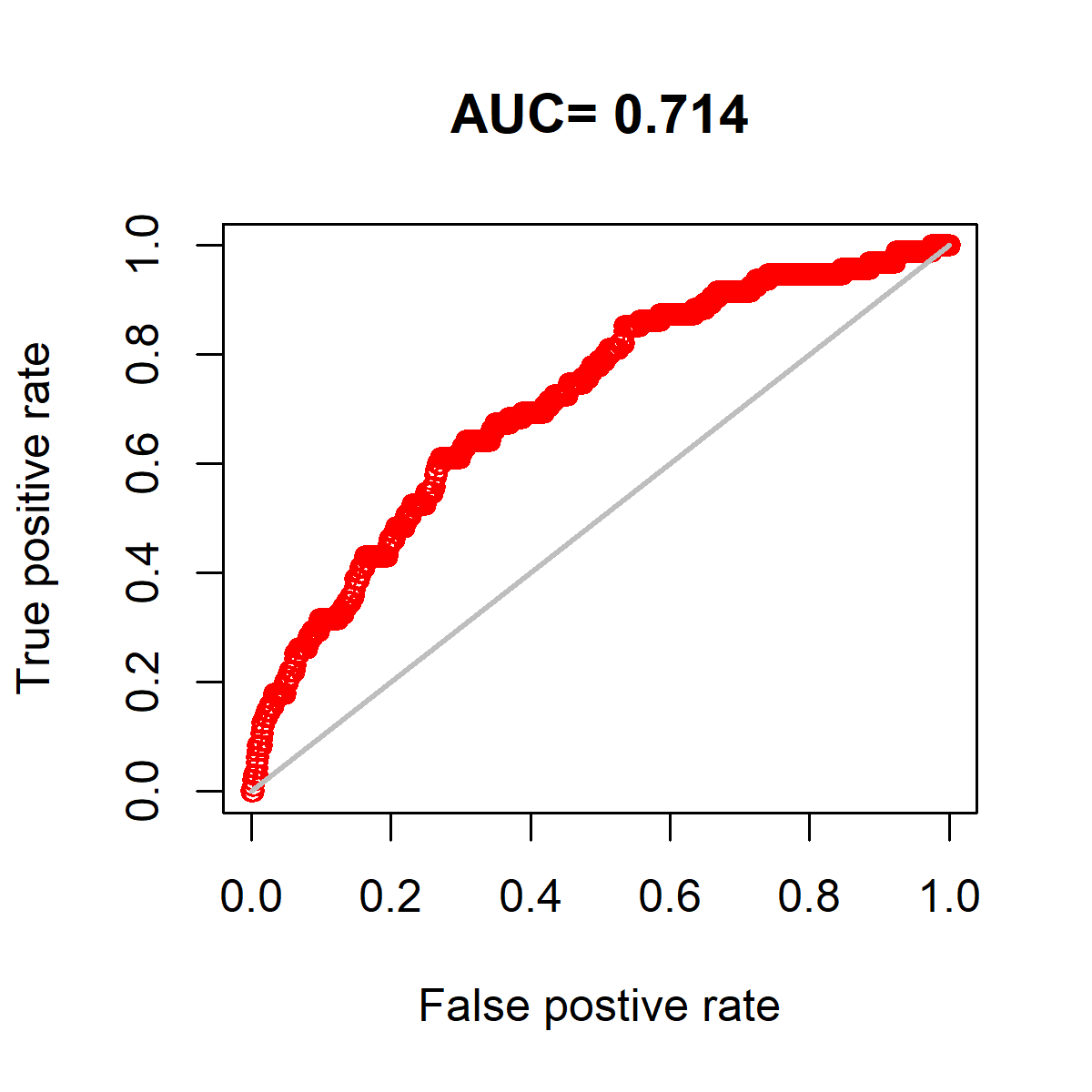


**Fig. S3**: The Area Under the Curve (AUC) of the Maxent model predicting the probability of herbivore occurrence at our study site.


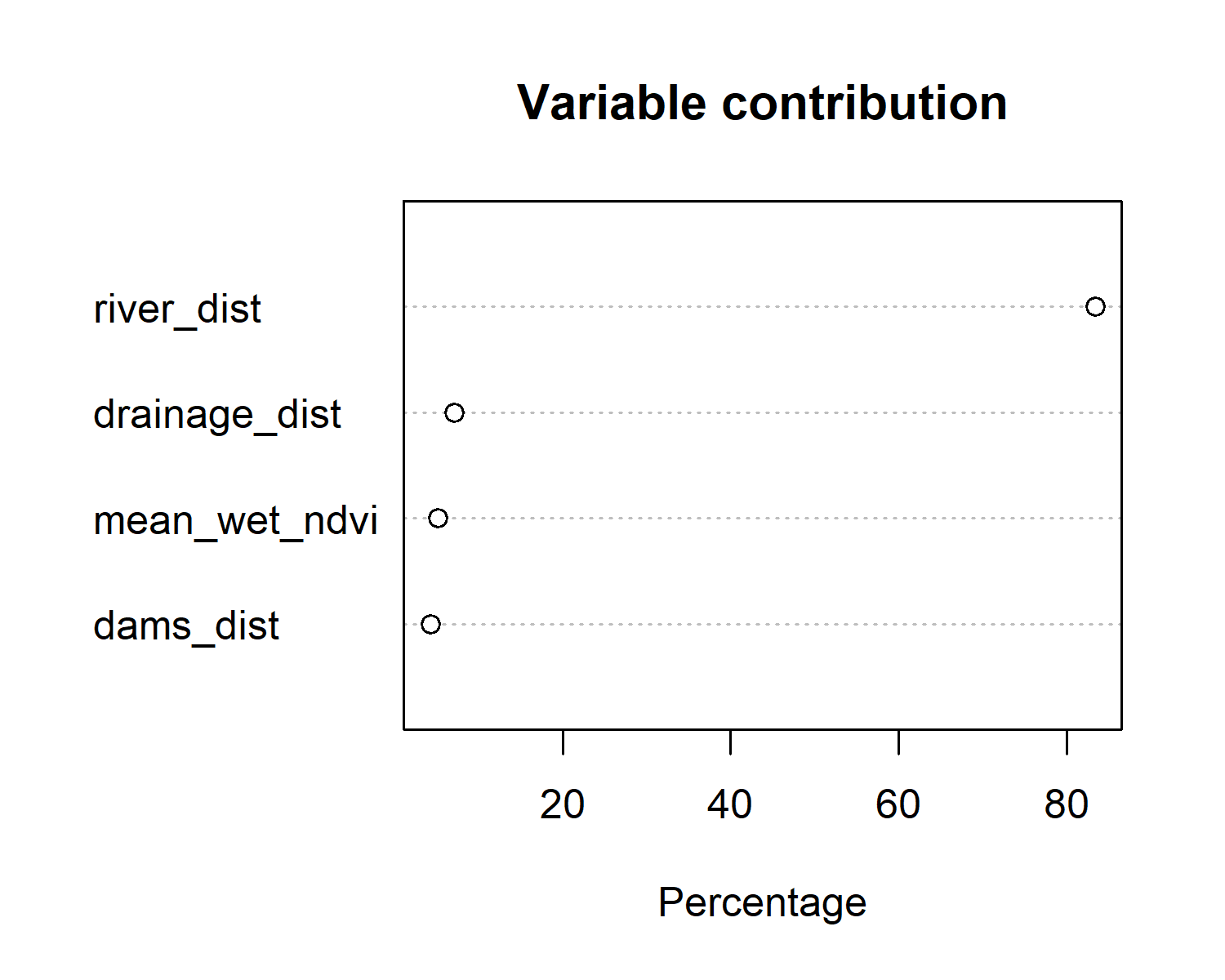


**Fig. S4:** The ranked importance for each of the environmental variables used in the Maxent model. River_dist refers to the distance away from rivers, drainage_dist refers to the distance away from drainage lines, mean_wet_ndvi refers to the mean NDVI values for 2015–2019, and dams_dist refers to the distance away from dams and pans. For differences in the contribution of each variable to the model (i.e., model using game trails and Maxent model) please see Table S2 listed below.


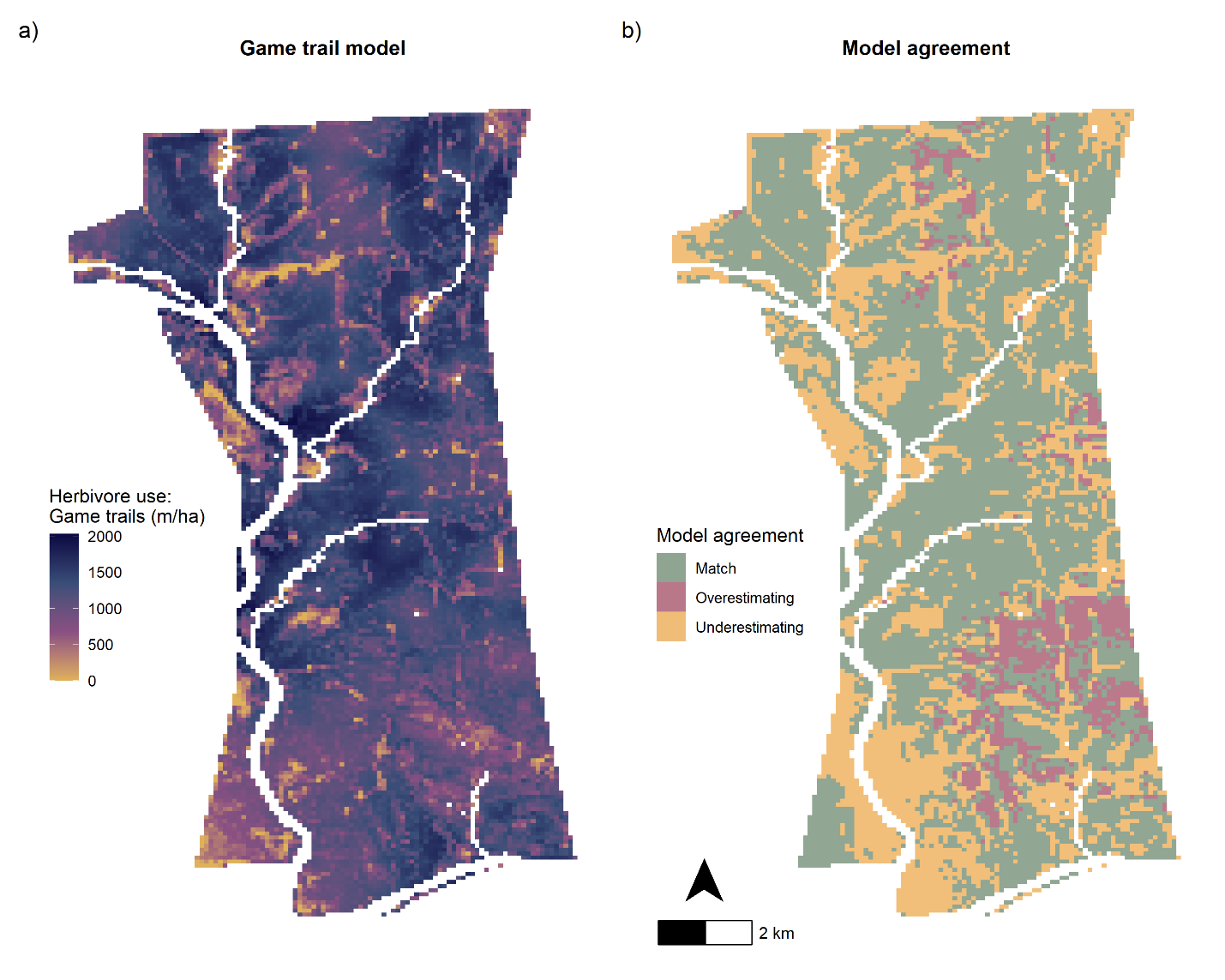


**Fig. S5:** Panel (a) shows the model-predicted patterns of herbivore habitat use across our study site from our model using the density of game trails (i.e., game trail model). Panel (b) shows model agreement between predicted patterns of herbivore habitat use between our game trail model and a Maxent model using the same environmental variables as our game trail model. Green represents areas where both models predicted the same level of herbivore habitat use across the landscape (~60%). Pink represents areas where our game trail model overestimated use compared the Maxent model (~8%), while yellow represents areas where our model underestimated use when compared to the Maxent model (~32%).

**Table S2**: Information-theoretic model selection process to select the best fit model to model the density of game trails at the landscape scale. Average NDVI and distance from dams were the smoothed variables whereas distance from drainage lines and distance from rivers were not smoothed.

| Model | Parameters | LogLik | AICc | ΔAICc | Weight |
| --- | --- | --- | --- | --- | --- |
| 1 | path_length ~ average NDVI + distance from river + distance from dams + distance from drainage lines | -1483.08 | 2995.417 | 0.000 | 0.600 |
| 2 | path_length ~ average NDVI + distance from river + distance from dams | -1485.09 | 2997.034 | 1.617 | 0.267 |
| 3 | path_length ~ average NDVI + distance from dams | -1485.91 | 2999.688 | 4.271 | 0.071 |
| 4 | path_length ~ average NDVI + distance from dams + distance from drainage lines | -1487.29 | 2999.995 | 4.578 | 0.061 |
| 5 | path_length ~ average NDVI + distance from river + distance from drainage lines | -1493.97 | 3008.316 | 12.899 | 0.001 |
| 6 | path_length ~ average NDVI + distance from river | -1496.53 | 3010.976 | 15.559 | 0.000 |
| 7 | path_length ~ average NDVI + distance from drainage lines | -1497.88 | 3014.548 | 19.131 | 0.000 |
| 8 | path_length ~ average NDVI | -1499.63 | 3015.601 | 20.184 | 0.000 |
| 9 | path_length ~ distance from river + distance from dams + distance from drainage lines | -1519.72 | 3056.991 | 61.574 | 0.000 |
| 10 | path_length ~ distance from river + distance from dams | -1521.03 | 3057.735 | 62.318 | 0.000 |
| 11 | path_length ~ distance from dams | -1525.18 | 3060.66 | 65.243 | 0.000 |
| 12 | path_length ~ distance from river + distance from drainage lines | -1524.05 | 3061.449 | 66.032 | 0.000 |
| 13 | path_length ~ distance from dams + distance from drainage lines | -1523.51 | 3062.302 | 66.885 | 0.000 |
| 14 | path_length ~ distance from river | -1527.15 | 3062.514 | 67.097 | 0.000 |
| 15 | path_length ~ 1 | -1529.73 | 3065.583 | 70.166 | 0.000 |
| 16 | path_length ~ distance from drainage lines | -1528.75 | 3065.705 | 70.288 | 0.000 |

**References:**

Abade, L., MacDonald, D. W., & Dickman, A. J. (2014). Using landscape and bioclimatic features to predict the distributions of lions, leopards and spotted hyaenas in Tanzania’s Ruaha Landscape. *PLoS ONE*, *9*, e96261.

Barnes, R. F. W. (2001). How reliable are dung counts for estimating elephant numbers? *African Journal of Ecology*, *39*, 1–9.

Burkepile, D. E., Burns, C. E., Tambling, C. J., Amendola, E., Buis, G. M., Govender, N., Nelson, V., Thompson, D. I., Zinn, A. D., & Smith, M. D. (2013). Habitat selection by large herbivores in a southern African savanna: The relative roles of bottom-up and top-down forces. *Ecosphere*, *4*, 1–19. https://doi.org/10.1890/ES13-00078.1

Elith, J., & Graham, C. H. (2009). Do they? How do they? WHY do they differ? On finding reasons for differing performances of species distribution models. *Ecography*, *32*, 66–77.

Estes, R. D. (1992). *The behavior guide to African mammals*. University of California Press.

Hijmans, R. J., Phillips, S. J., Leathwick, J., & Elith, J. (2021). *Dismo: Species Distribution Modeling. R package version 1.3-5. Https://CRAN.R-project.org/package=dismo* [Computer software]. https://CRAN.R-project.org/package=dismo

Leeuwis, T., Peel, M. J. S., & De Boer, W. F. (2018). Complexity in African savannas: Direct, indirect, and cascading effects of animal densities, rainfall and vegetation availability. *PLoS ONE*, *13(5)*, e0197149.

Liu, C., Newell, G., & White, M. (2016). On the selection of thresholds for predicting species occurrence with presence-only data. *Ecology and Evolution*, *6*, 337–348.

Matawa, F., Murwira, A., & Schmidt, K. S. (2012). Explaining elephant (Loxodonta africana) and buffalo (Syncerus caffer) spatial distribution in the Zambezi Valley using maximum entropy modelling. *Ecological Modelling*, *242*, 189–197.

Merow, C., Smith, M. J., & Silander, J. A. (2013). A practical guide to MaxEnt for modeling species’ distributions: What it does, and why inputs and settings matter. *Ecography*, *36*, 1058–1069.

Phillips, S. J., Anderson, R. P., & Schapire, R. E. (2006). Maximum entropy modeling of species geographic distributions. *Ecological Modelling*, *190*, 231–259. https://doi.org/10.1016/j.ecolmodel.2005.03.026

Phillips, S. J., & Dudík, M. (2008). Modeling of species distributions with Maxent: New extensions and a comprehensive evaluation. *Ecography*, *31*, 161–175.

Schmitt, M. H., Stears, K., Donovan, M. K., Burkepile, D. E., & Thompson, D. I. (2022). Integrating herbivore assemblages and woody plant cover in an African savanna to reveal how herbivores respond to ecosystem management. *PLoS ONE*, *17*(8), e0273917. https://doi.org/10.1371/journal.pone.0273917

Skinner, J. D., & Chimimba, C. T. (2005). *The mammals of the southern African subregion* (Third). Cambridge University Press.

Su, H., Bista, M., & Li, M. (2021). Mapping habitat suitability for Asiatic black bear and red panda in Makalu Barun National Park of Nepal from Maxent and GARP models. *Scientific Reports*, *11*, 14135.

Zhang, J., Jiang, F. J., Li, G., Qin, W., Li, S., Gao, H., Cai, Z., Lin, G., & Zhang, T. (2019). Maxent modeling for predicting the spatial distribution of three raptors in the Sanjiangyuan National Park, China. *Ecology and Evolution*, *9*, 6643–6654.

Zuur, A. F., Ieno, E. N., Walker, N. J., Saveliev, A. A., & Smith, G. M. (2009). *Mixed effects models and extensions in ecology with R.* Springer.
